# Psychological changes among women with recurrent pregnancy loss during the COVID-19 period in northeastern China: a cross-sectional study

**DOI:** 10.3389/fpsyg.2023.1265926

**Published:** 2023-10-23

**Authors:** Tingting Wang, Yue Hou, Yilin Liu, Chong Qiao

**Affiliations:** ^1^Department of Obstetrics and Gynecology, Shengjing Hospital of China Medical University, Shenyang, China; ^2^Key Laboratory of Obstetrics and Gynecology of Higher Education of Liaoning Province, Shenyang, China

**Keywords:** COVID-19, recurrent pregnancy loss, anxiety, depression, social support, stress

## Abstract

**Background:**

It aimed to investigate the prevalence of anxiety and depression in recurrent pregnancy loss (RPL) women and the related factors in Northeastern China during the Coronavirus disease 2019 (COVID-19) pandemic.

**Methods:**

From March to June 2021, we conducted an electronic questionnaire survey of 267 RPL women who attended the recurrent pregnancy loss clinic at Shengjing Hospital of China Medical University. The State–Trait Anxiety Inventory (STAI), Beck Depression Inventory-II (BDI-II), Perceived Stress Scale (PSS), and Medical Outcomes Study Social Support Survey (MOS-SSS-C) were used to screen for anxiety, depression, stress, and social support. Logistic regression was used to explore the related factors of anxiety and depression.

**Results:**

RPL women had severe psychological problems during the pandemic: 56.6% showed state anxiety or trait anxiety, 26.6% showed high levels of stress, and 13.1% showed depression. Economic pressure caused by COVID-19, and high stress were common related factors for anxiety and depression. The interval since last miscarriage <6 months, worse mood changes due to COVID-19, and concerns about COVID-19 were associated with anxiety. A history of pregnancy loss >14 weeks was associated with depression. While adequate social support and actively seeking health help were protective factors for trait anxiety. And identifying the etiology was a protective factor for depression.

**Conclusion:**

The study revealed the prevalence of anxiety, depression, and the associated factors in RPL women during COVID-19. More attention should be paid to the psychology of RPL women and adequate social support should be provided.

## Introduction

COVID-19 is the largest pandemic affecting global public health after severe acute respiratory syndrome (SARS) in 2002. The rapid emergence of new viral phenotypes (Kannan et al., 2021), sporadic reports of breakthrough infections (Bergwerk et al., 2021), and repeated outbreaks pose a huge chanllenge for ending the pandemic.

Previous studies have shown that a pandemic of infectious diseases can lead to mental health abnormalities in populations, ranging from mood disorders, mental stress, anxiety, and depression to psychosis and cognitive impairment (Chua et al., 2004; Rubin et al., 2010). Investigations revealed that COVID-19 led to a 27.6% increase in depression cases and a 25.6% increase in anxiety cases globally (Daly and Robinson, 2022). Pregnant women are at heightened risk of infections with the virus. Virus infection causes poor prognosis for the fetus and mother and increases anxiety and depression levels. A study revealed that the incidence of depression in pregnant women was 35.4% during COVID-19 (Durankuş and Aksu, 2022). Epidemic-control measures on health management measures, confinement, and social distancing may have an impact on antepartum, expectant, and postpartum health (Kotlar et al., 2021; Motrico et al., 2021). In conclusion, the differences among these studies are due to population differences, the severity of COVID-19, and socioeconomic differences.

However, it’s still unknown what the psychological changes of a certain population are. Recurrent pregnancy loss (RPL) refers to two or more spontaneous miscarriages (EL Hachem et al., 2017), including biochemical pregnancies in our study. During a pandemic, RPL women faced concerns about fertility issues in addition to fear of infection, economic pressure, family members’ infection, their health and infants’, and future uncertainties (e.g., successful pregnancy preparation, maintenance of pregnancy, and delivery process). Furthermore, prenatal anxiety and depression are not only connected with obstetric complications such as premature delivery (Grigoriadis et al., 2018), and miscarriage (Qu et al., 2017), but also with changes to brain structure and function (Adamson et al., 2018), and cognitive impairment (Davis and Sandman, 2010) in infants. Therefore, the mental health problems of RPL women are extremely important.

Our research group began to focus on outbreak prevention and control measures at the early stage of the pandemic in RPL women. We took the lead in compiling and publishing guidelines for the management of pregnant women during COVID-19 nationwide and started a remote online consultation system in March 2021, which basically meets the needs of RPL women at different stages. Despite this, RPL women still have certain psychological problems. Thus, a variety of scales were used to comprehensively evaluate the psychology of RPL women, and socio-demographic information, specific adverse maternal history, and the impact of COVID-19 were integrated.

Based on a cross-sectional study in Northeastern China, we analyzed the prevalence of anxiety, depression, and the related factors in RPL women during COVID-19, to provide clinical evidence for psychological intervention in RPL women.

## Materials and methods

### Study design and population inclusion

This was a cross-sectional study conducted in Northeastern China. A total of 267 women who attended the recurrent pregnancy loss clinic at Shengjing Hospital of China Medical University from March 2021 to June 2021 were enrolled. Women aged 20–45 with no history of psychiatric illness or use of psychiatric medications, and willing to accept and cooperate with the survey were eligible to participate. Women with a history of cognitive or psychiatric disorders and those taking any psychotropic medications were excluded. According to the different stages of pregnancy, 267 included RPL women were in the following stages: not preparing for pregnancy (84 cases), preparing for pregnancy (87 cases), the first trimester (23 cases), the second trimester (53 cases), and the third trimester (11 cases), antepartum (4 cases) and postpartum (5 cases). All women gave informed consent to participate.

An online-based survey program “Questionnaire Star” was used to collect data. The structured questionnaire includes several aspects:

### Psychological questionnaires

#### State–trait anxiety inventory

State–trait anxiety inventory (STAI) was used to intuitively reflect the subjective feelings of anxious patients. The scale consists of two subscales with a total of 40 questions, which evaluate two different types of anxiety. The S-AI subscale consists of the first 20 questions, which reflect the subject’s current anxiety (state anxiety). The T-AI subscale consists of the last 20 questions, which reflect the subject’s usual anxiety (trait anxiety). Both scales scored between 20 and 80. The higher the score, the higher the anxiety. 40 was used as a cut-off value for state anxiety and trait anxiety (Julian, 2011). Good reliability and validity were demonstrated in the Chinese version of the scale (Du et al., 2022).

#### Beck depression inventory-II

Beck depression inventory-II (BDI-II) was used to assess depression levels in psychiatric patients and the general population (Smarr and Keefer, 2011). 16 is considered to be a clinically significant threshold for identifying depression, and the sensitivity is 88.2%, and the specificity is 92.1% (Huffman et al., 2010). The Chinese version of the BDI-II possessed acceptable psychometric properties (Shek, 1990). And one study reported that the Chinese version of BDI-II has a good reliability of 0.88 to 0.92 among Chinese middle school teachers (Wang et al., 2020).

#### Perceived stress scale

The 14-item Perceived stress scale (PSS) was used to assess women’s perceived stress. Items were rated according to a five-point Likert-type scale. The higher the score, the more stress you feel. The Chinese version had good internal consistency, with alpha values of 0.74 to 0.91 (Huang et al., 2020; She et al., 2021). We used PSS score quartiles to quantify stress, and 29 was used as a cut-off value in this study (Polinski et al., 2020).

#### Medical outcomes study social support survey (MOS-SSS-C)

Mainly measuring social support function, this scale is a non-self-administered questionnaire that includes four dimensions: Affectionate support, Tangible support, Positive social interaction support, and Emotional/informational support. Higher scores indicate higher levels of support received. The alpha value of the Chinese version was 0.98 and the test–retest reliability was 0.84, indicating the scale’s stable application (Yu et al., 2004).

#### COVID-19 questionnaire

Participants completed a questionnaire about the impact and perception of COVID-19. The content includes knowledge about COVID-19 (e.g., source, effective protective measures), attitudes toward COVID-19 (e.g., the control time, the development trend), the impact of COVID-19 (e.g., economy, life), and demand during COVID-19 (e.g., medical needs).

#### Socio-demographic information and obstetric characteristics

Age, BMI, education attainment, monthly household income (yuan), stage of diagnosis and treatment, stage of pregnancy, live birth, and history of abnormal pregnancy were included.

### Statistical analysis

The average was used to describe the score of each scale. Student’s t test or One-way ANOVA was used for continuous variables. The chi-square (*χ*^2^) test was used for categorical variables. The correlation between social support and stress, anxiety, depression was analyzed by Pearson correlation analysis. The potential related factors for anxiety and depression were analyzed by Logistic regression analysis. *p* < 0.05 was considered statistically significant. Statistical Package for the Social Sciences 25.0 (SPSS 25.0) software was used to analyze the data.

## Results

### Socio-demographic information and obstetric characteristics

The basic information and psychological scale scores of RPL women are presented in Table 1. For socio-demographic information, the average age of the participants was 33.46. 84 of the 267 study participants were screening for the etiology and 183 have identified the etiology. For obstetric characteristics, 4.5% of participants had a history of live birth, 9.4% had a history of pregnancy loss after 14 weeks, and 89.9% had two or three miscarriages. The interval between filling out the form and last miscarriage was more than 6 months in most RPL women (77.2%). For psychological status, 56.6% of RPL women showed state anxiety, 56.6% showed trait anxiety, 13.1% showed depression, 26.6% showed high stress, and 10.5% showed low social support.

**Table 1:** Socio-demographic information, obstetric characteristics and psychological scale scores of RPL women (*n* = 267).

Variables	*N* (%) / Mean (SD)
Age	33.46 (3.528)
Education	
Primary school and junior high school	27 (10.1)
Senior Secondary and Junior College	75 (28.1)
Bachelor’s degree or above	165 (61.8)
Monthly household income (yuan)	
<5,000	82 (30.7)
5,000–9,999	143 (53.6)
≥10,000	42 (15.7)
Stage of diagnosis and treatment	
Screening for the etiology	84 (31.5)
Identifying the etiology	183 (68.5)
Live birth	
Yes	12 (4.5)
No	255 (95.5)
Pregnancy loss > 14 weeks	
Yes	25 (9.4)
No	242 (90.6)
Number of lost pregnancies	
2–3	240 (89.9)
≥4	27 (10.1)
Interval since last miscarriage (months)	
<6	61 (22.8)
≥6	206 (77.2)
S-AI	
<40	116 (43.4)
≥40	151 (56.6)
T-AI	
<40	116 (43.4)
≥40	151 (56.6)
BDI-II	
0–9 Health	184 (68.9)
10–15 Mild emotional distress	48 (18.0)
16–24 Depression	20 (7.5)
≥25 Severe depression	15 (5.6)
PSS	
<29 Low and moderate stress	196 (73.4)
≥29 High stress	71 (26.6)
MOS-SSS-C	
<50 Low social support	28 (10.5)
≥50 High social support	239 (89.5)

S-AI, state anxiety; T-AI, trait anxiety; BDI-II, Beck depression inventory-II; PSS, perceived stress scale; MOS-SSS-C, medical outcomes study social support survey.

### Knowledge about COVID-19, attitudes toward COVID-19, and the impact of COVID-19

**Figure 1:**
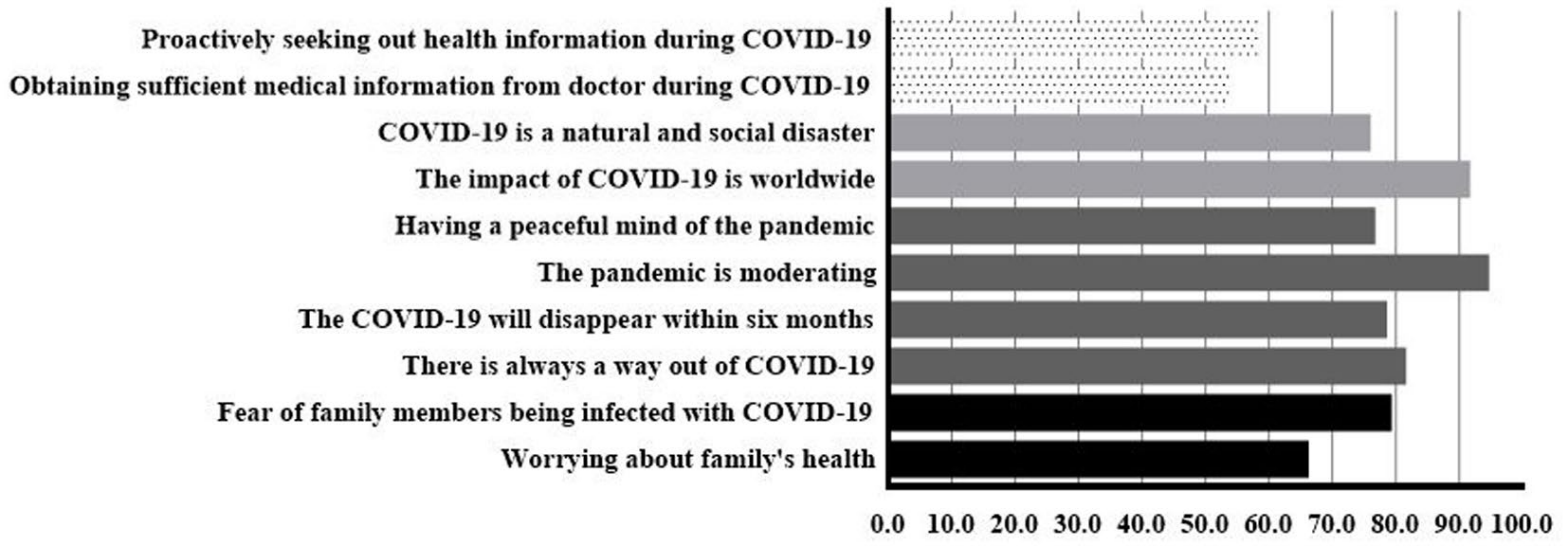
Worry about COVID-19 (black), attitudes toward COVID-19 (dark grey), knowledge about COVID-19 (light grey), demand during COVID-19 (dot).

As shown in Figure 1, information about COVID-19 in RPL women was collected. For knowledge about COVID-19, 76.03% of RPL women believed that COVID-19 was a natural and social disaster, and 91.76% held that the impact of COVID-19 was worldwide. For attitudes toward COVID-19, 94.76% thought the pandemic is moderating. 81.65% thought that there is always a way out of COVID-19. 78.65% thought COVID-19 could disappear within 6 months. 76.78% hold a peaceful mind. For worry about COVID-19, more than half of RPL women worried about their families. For demand during COVID-19, medical information seems to be important. We also investigated the hygiene practices changes of RPL women during COVID-19. Almost everyone has practiced good hygiene during COVID-19. Overall, the average hygiene score during COVID-19 was 12.64. We found that 89.1% of RPL women had good hygiene practices during COVID-19 (Supplementary Table S1).

### Psychological changes in different stages of RPL women

**Figure 2:**
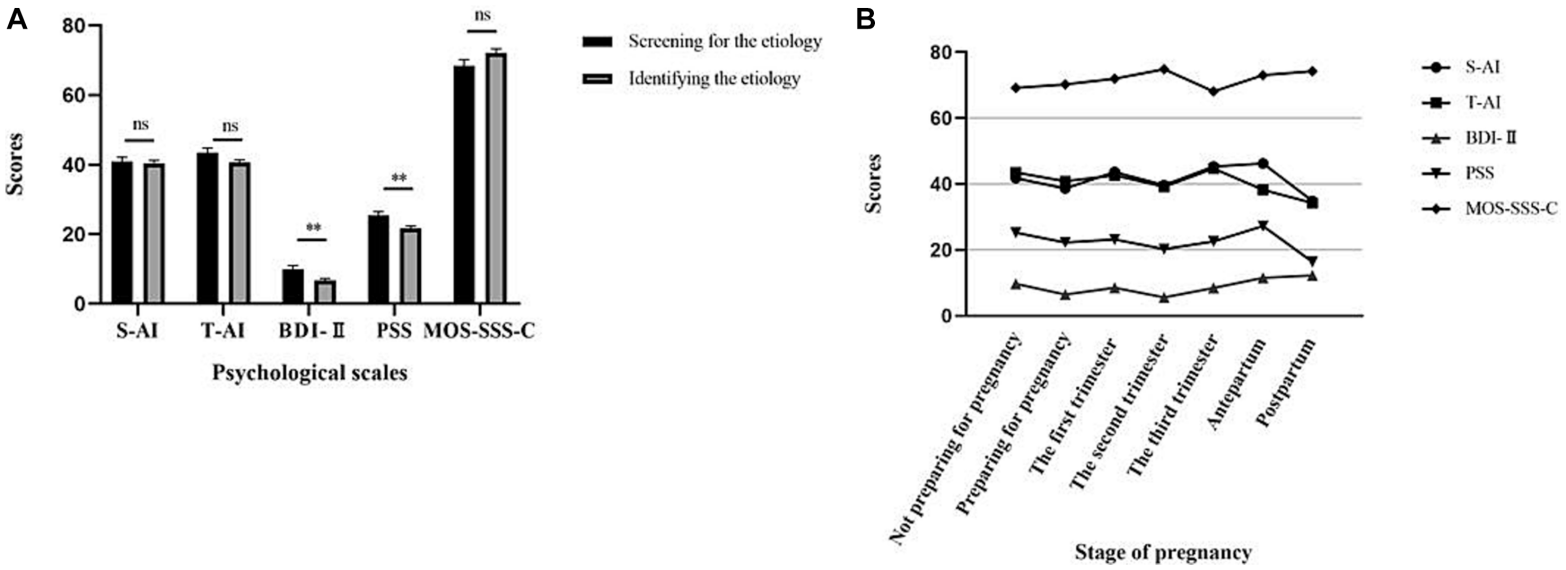
Psychological changes in different stages in RPL women. **(A)**. Anxiety (S-AI/T-AI), depression (BDI-II), stress (PSS), and social support (MOS-SSS-C) scores of RPL women at different stages of diagnosis and treatment. **(B)**. Anxiety (S-AI/T-AI), depression (BDI-II), stress (PSS), and social support (MOS-SSS-C) scores of RPL women at different stages of pregnancy. S-AI, state anxiety; T-AI, trait anxiety; BDI-II, Beck depression Inventory-II; PSS, perceived stress scale; MOS-SSS-C, medical outcomes study social support survey.

As shown in Figure 2A, according to the process of disease diagnosis and treatment, participants were divided into two groups: those who were screening for the etiology and those who had completed etiological screening and identified the etiology of miscarriage. The comparative score showed that after completing etiological screening and identifying the etiology, the level of stress, anxiety, and depression in RPL women decreased, especially scores of depression (*p* = 0.003) and stress (*p* = 0.004) reduced significantly, while the level of social support increased.

We further compared psychological changes in different stages of pregnancy. We noted a lower level of stress, anxiety, and depression after receiving permission to prepare for pregnancy. With a successful pregnancy, there was an upward trend in negative emotions, which might be related to concern about another miscarriage. The emotion is gradually relieved as the pregnancy progresses, reaching a peak again before delivery and leveling off in the postpartum period. There was a significant reduction in stress (*p* = 0.046) and in depression levels (*p* = 0.012) between the stage of not preparing for pregnancy and the stage of preparing for pregnancy. No statistically significant differences were found between the remaining two adjacent phases. The trend of each psychological score with the stage of pregnancy is presented in Figure 2B.

### Correlation between social support and stress, anxiety, depression

The MOS-SSS-C scale was first applied to RPL women, and reliability analysis showed good reliability in RPL women, with a Cronbach α coefficient of 0.961 and a folded half coefficient of 0.986 (Supplementary Table S2). The reliability coefficient > 0.8 indicates that the social support scale has high consistency, reliability, and stability in RPL women.

Pearson correlation analysis showed that stress, anxiety, and depression were negatively correlated with both total social support and each dimension (*p* < 0.001), as shown in Table 2.

**Table 2:** Associations between social support and stress (PSS), anxiety (S-AI/T-AI), depression (BDI-II).

Social support subscale	PSS		S-AI		T-AI		BDI-II	
	*r*	*p* value	*r*	*p* value	*r*	*p* value	*r*	*p* value
Total support	−0.439	<0.001^***^	−0.372	<0.001^***^	−0.436	<0.001^***^	−0.390	<0.001^***^
Affectionate support	−0.334	<0.001^***^	−0.288	<0.001^***^	−0.335	<0.001^***^	−0.234	<0.001^***^
Tangible support	−0.242	<0.001^***^	−0.174	<0.001^***^	−0.250	<0.001^***^	−0.119	<0.001^***^
Positive social interaction support	−0.427	<0.001^***^	−0.345	<0.001^***^	−0.403	<0.001^***^	−0.319	<0.001^***^
Emotional/ informational support	−0.385	<0.001^***^	−0.331	<0.001^***^	−0.359	<0.001^***^	−0.272	<0.001^***^

^*^p < 0.05.

^**^p < 0.01.

^***^p < 0.001.

S-AI, state anxiety; T-AI, trait anxiety; BDI-II, Beck depression inventory-II; PSS, perceived stress scale.

### Factors associated with anxiety, and depression

**Figure 3:**
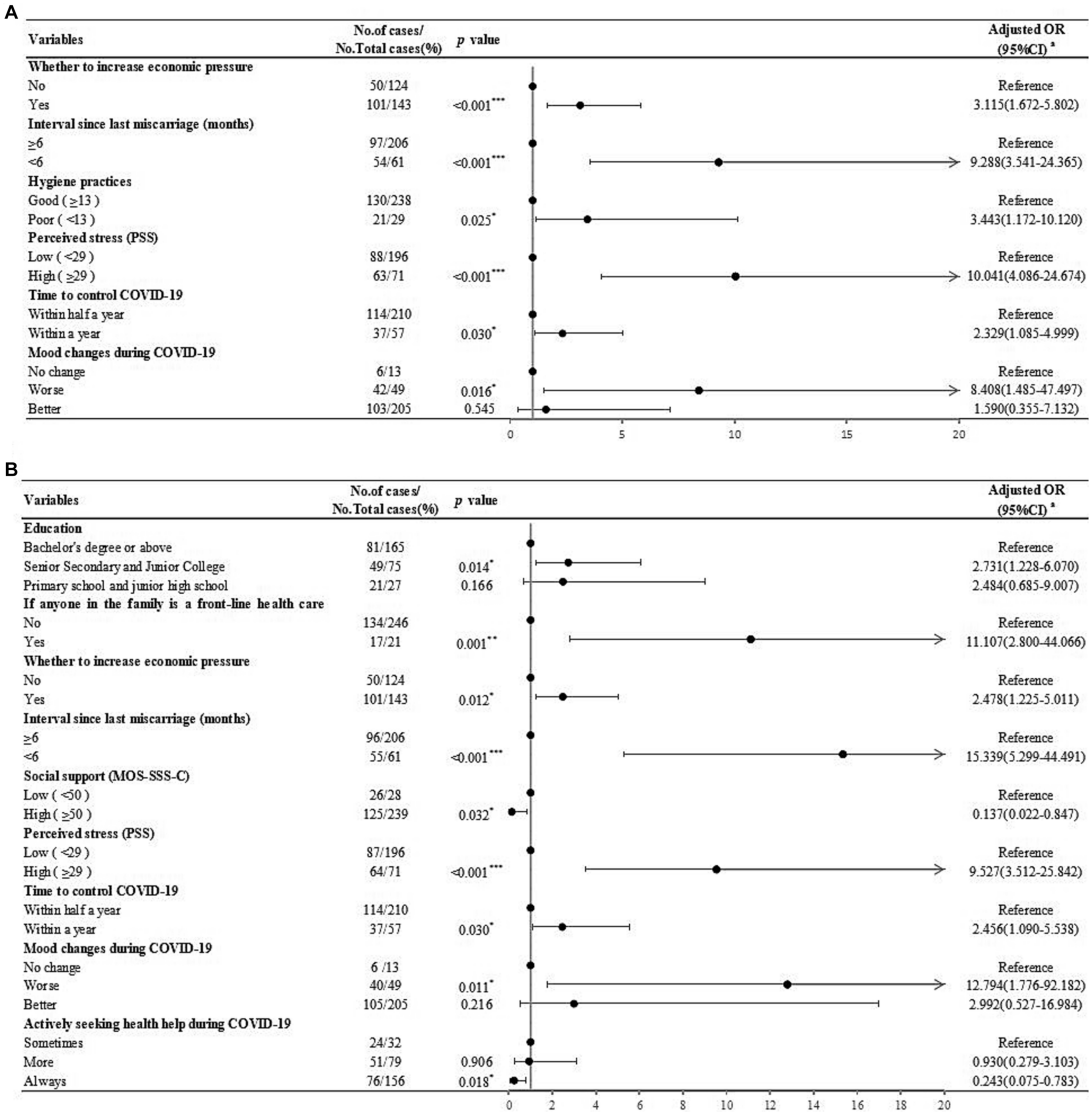
Risk factors for anxiety identified by logistic regression analysis in RPL women. **(A)**. Risk factors for state anxiety (S-AI scores ≥40) identified by logistic regression analysis in RPL women. **(B)**. Risk factors for trait anxiety (T-AI scores ≥40) identified by logistic regression analysis in RPL women.^a^Adjusted for age, BMI, location, knowledge, attitude, worries regarding COVID-19, and behavioral changes since COVID-19. ^*^p < 0.05; ^**^p < 0.01; ^***^p < 0.001.

After adjusting for relevant confounders, increased economic pressure caused by COVID-19 (aOR = 3.115; 95%CI = 1.672, 5.802; *p* < 0.001), the interval since last miscarriage <6 months (aOR = 9.288; 95%CI = 3.541, 24.365; *p* < 0.001), poor hygiene practices (aOR = 3.443; 95%CI = 1.172, 10.120; *p* = 0.025), high stress (aOR = 10.041; 95%CI = 4.086, 24.674; *p* < 0.001), thinking it will take a year to control COVID-19 (aOR = 2.329; 95%CI = 1.085, 4.999; *p* = 0.03), and worse mood changes during COVID-19 (aOR = 8.408; 95%CI = 1.485, 47.497; *p* = 0.016) are associated with state anxiety in RPL women (Figure 3A). Senior Secondary and Junior College education (aOR = 2.731; 95%CI = 1.228, 6.070; *p* = 0.014), having a front-line medical worker in the family (aOR = 11.107; 95%CI = 2.800, 44.066; *p* = 0.001), increased economic pressure caused by COVID-19 (aOR = 2.478; 95%CI = 1.225, 5.011; *p* = 0.012), the interval since last miscarriage <6 months (aOR = 15.339; 95%CI = 5.299, 44.491; *p* < 0.001), thinking it will take a year to control COVID-19 (aOR = 2.456; 95%CI = 1.090, 5.538; *p* = 0.030), worse mood changes during COVID-19 (aOR = 12.794; 95%CI = 1.776, 92.182; *p* = 0.011) and high stress (aOR = 9.527; 95%CI = 3.512, 25.842; *p* < 0.001) are associated with trait anxiety in RPL women. While adequate social support (aOR = 0.137; 95%CI = 0.022, 0.847; *p* = 0.032) and actively seeking health help (aOR = 0.243; 95%CI = 0.075, 0.783; *p* = 0.018) are protective factors for trait anxiety in RPL women (Figure 3B).

**Figure 4:**
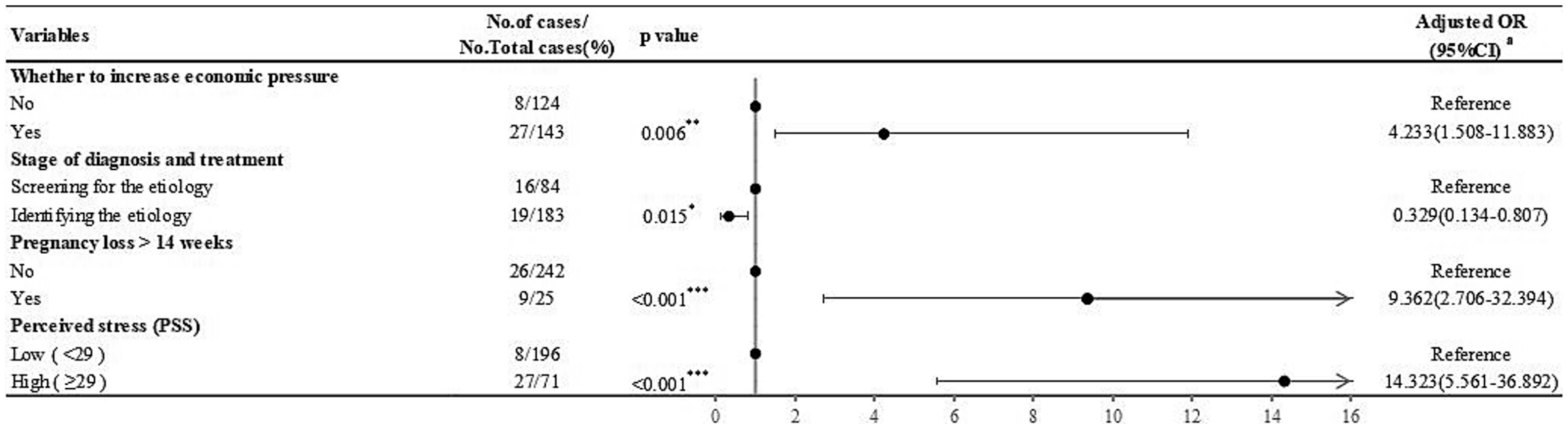
Risk factors for depression (BDI-II scores ≥16) identified by logistic regression analysis in RPL women. ^a^Adjusted for age, BMI, location, knowledge, attitude, worries regarding COVID-19, and behavioral changes during COVID-19. ^*^p < 0.05;^**^p < 0.01;^***^p < 0.001.

Similarly, after adjusting for relevant confounders, increased economic pressure caused by COVID-19 (aOR = 4.233; 95%CI = 1.508, 11.883; *p* = 0.006), a history of pregnancy loss >14 weeks (aOR = 9.362; 95%CI = 2.706, 32.394; *p* < 0.001), and high stress (aOR = 14.323; 95%CI = 5.561, 36.892; *p* < 0.001) are associated with depression in RPL women. While completing etiological screening and identifying the etiology (aOR = 0.329; 95%CI = 0.134, 0.807; *p* = 0.015) was a protective factor for depression in RPL women (Figure 4).

## Discussion

The present study revealed the psychological state of RPL women in Northeastern China during COVID-19. COVID-19 is considered to be even more of a significant stressor. It has a negative impact on the mental health of vulnerable or high-risk populations such as adolescents (Commodari and La Rosa, 2020), young adults (La Rosa and Commodari, 2023), and pregnant women (Durankuş and Aksu, 2022). RPL women are faced with the uncertainty of pregnancy maintenance and the possibility of another miscarriage and are prone to psychological problems. To the best of our knowledge, this is the first study to explore the psychological state and the related factors of RPL women during COVID-19. According to the findings, there was a considerable degree of anxiety, and depression among RPL women during COVID-19. This suggests that attention should be paid to the psychological status of RPL women during a disease pandemic.

In the progression of various chronic diseases, there are differences in patients’ psychology at different stages, such as treatment after diagnosis, preoperatively, postoperatively, and during chemotherapy. However, few people have explored the psychological fluctuations of RPL women at different stages of diagnosis and treatment. We found that in RPL women who have identified the etiology, their stress, and depression levels decreased significantly. The possible reason is that at the beginning of treatment, the unclear etiology and the success of pregnancy preparation can be irritating factors that affect the psychology of RPL women (Richardson et al., 2017). RPL women face different problems, from the uncertainty of the etiology and the success of pregnancy preparation to the maintenance of pregnancy and successful delivery. They need to be psychologically strong in addition to taking on new responsibilities and roles. As research has shown (Qu et al., 2021), social support, anxiety, and depression changed with the trimesters in pregnant RPL women. At the same time, looking at the whole process of RPL women from preparation for pregnancy to delivery, we noted that not preparing for pregnancy, the first trimester, the third trimester and the antepartum period are the four more psychologically vulnerable stages for RPL women. Given the high incidence of first-trimester miscarriage, early pregnancy is a period of mental stress for these women, and thus they are prone to anxiety and depression. Similarly, worries about the fetus and self in the third trimester and antepartum period are also likely to cause anxiety and depression. In response to this finding, we should implement a step-wise intervention for RPL women at different stages of pregnancy, focusing on the four periods of relative psychological vulnerability. That is, standardizing diagnosis and treatment in the stage of not preparing for pregnancy, strengthing health care in the first trimester, and providing adequate social support in the third trimester and antepartum period.

Associated socio-demographic information and obstetric characteristics among RPL women during COVID-19 have also been assessed. We found that low education was associated with trait anxiety. Probably due to RPL women with low education had no clear perception of the disease and had difficulty complying with medical advice. And a history of pregnancy loss >14 weeks was associated with depression. Miscarriage in the first trimester has a child who is not fully developed, however, after the second trimester, with the gradual feeling of fetal heart and fetal movement, psychological pressure also gradually increases (Couto et al., 2009). Beyond that, the interval since last miscarriage <6 months was associated with anxiety. This is consistent with the previous study, that a significant proportion of women show distress shortly after miscarriage, but the level of distress decreases over time (Lok et al., 2010).

The findings also showed that COVID-19 related life changes, with attitudes toward COVID-19 had a moderate but significant impact on the occurrence of negative emotions. Specifically, a worse mood change and negative attitude during COVID-19 seemed to be associated with anxiety. And increased economic pressure caused by COVID-19 was a related factor for depression and trait anxiety in RPL women. The possible mechanism by which stressful economic events lead to mental changes is related to the activated inflammatory response and endocrine system (Bruckner et al., 2016). Conversely, maintaining good hygiene practices, and actively seeking and obtaining adequate help from a physician were protective factors for anxiety. RPL women expect healthcare providers to show understand, listen, and concern to them. However, the closure of healthcare institutions during the pandemic prevented women from accessing time-sensitive examinations and health care (Kotlar et al., 2021). Therefore, it is important to provide health care actively and foster a good doctor-patient relationship to help RPL women during COVID-19. As the former study found, high social support contributes to mental health of women during pregnancy (Friedman et al., 2020). Our study revealed that adequate social support was a protective factor for trait anxiety and identifying the etiology was a protective factor for depression. Given these insights, possible policies to promote the mental health of RPL women during the pandemic are promoting healthy habits and lifestyles, reducing economic stress, relieving mental stress, increasing dispositional positivity, fostering a good doctor-patient relationship, and providing adequate social support.

### Limitations and strengths

Several limitations arise in this study. First, this was a cross-sectional study, so the exact causal relationship between variables cannot be determined. Second, the sample size included in this research was small, but it met the minimum sample size. We will continue to collect data and conduct follow-up studies in the future. However, despite these limitations, our study demonstrated the timely psychological response to COVID-19 and periodic psychological changes of RPL women and explored the related factors. It will guide the formulation of psychological support strategies for RPL women in China and other epidemy-affected regions.

## Conclusion

This study reported a significant prevalence of anxiety and depression in RPL women during COVID-19. Increased economic pressure caused by COVID-19 and worry about COVID-19, together with the history of adverse pregnancy, were associated with anxiety and depression in RPL women. Identification of the etiology, adequate social support, and actively seeking health help appeared to be protective factors for negative emotions in RPL women. Therefore, it is necessary to develop appropriate strategies, pay close attention to the stage-specific psychological changes of RPL women, implement step-wise intervention for RPL women’s psychology, standardize the diagnosis and treatment, and give adequate social support to promote mental health.

## Data availability statement

The original contributions presented in the study are included in the article/Supplementary material, further inquiries can be directed to the corresponding author.

## Ethics statement

This study was examined and approved by the Ethics Committee of China Medical University (approval number: 2018PS381K).

## Author contributions

TW: Investigation, Methodology, Writing – original draft, Writing – review & editing. YH: Supervision, Writing – review & editing. YL: Methodology, Writing – review & editing. CQ: Funding acquisition, Writing – review & editing.

## Abbreviations

COVID-19: Coronavirus 2019
RPL: Recurrent pregnancy loss
STAI: State–trait anxiety inventory
S-AI: State anxiety
T-AI: Trait anxiety
BDI-II: Beck depression inventory-II
PSS: Perceived stress scale
MOS-SSS-C: Medical outcomes study social support survey
