# Supplementary material for: Psychological changes among women with recurrent pregnancy loss during the COVID-19 period in northeastern China: a cross-sectional study
Source: Front Psychol. 2023 Oct 23;14:1265926. doi: 10.3389/fpsyg.2023.1265926 (PMC10626446; doi:10.3389/fpsyg.2023.1265926)
Supplement: Supplementary file 1 [file Table_1.DOC]

**Supplementary table 1.** **Hygiene practices of RPL women during COVID-19**

| **Hygiene practices^#*^** | Mean (SD) | **N (%)** |
| --- | --- | --- |
| Wash hands before meals |  | 259 (97) |
| Wash hands after using the toilet |  | 258 (96.6) |
| Cover the mouth when coughing and sneezing |  | 261 (97.7) |
| Open window for ventilation |  | 222 (83.1) |
| **Total score (0-20)** | 12.64 (1.606) |  |
| **Hygiene Sub-section** |  | **N (%)** |
| Poor (<13) |  | 29 (10.9) |
| Good (≥13) |  | 238 (89.1) |

^#^Indicates those whose frequency is more or always

^*^Hygiene measured on Likert Scale where 1 never - 5 always

**Supplementary table 2. Reliability analysis of medical support scale (MOS-SSS-C)**

| **Reliability analysis** | **Cronbach α coefficient** | **Folded half coefficient** |
| --- | --- | --- |
| **Total support** | 0.961 | 0.986 |
| **Tangible support** | 0.844 | 0.870 |
| **Emotional/informational support** | 0.932 | 0.981 |
| **Positive social interaction support** | 0.852 | 0.851 |
| **Affectionate support** | 0.872 | 0.827 |
